# Cathepsin K: The Action in and Beyond Bone

**DOI:** 10.3389/fcell.2020.00433

**Published:** 2020-06-04

**Authors:** Rongchen Dai, Zeting Wu, Hang Yin Chu, Jun Lu, Aiping Lyu, Jin Liu, Ge Zhang

**Affiliations:** ^1^Law Sau Fai Institute for Advancing Translational Medicine in Bone & Joint Diseases, Hong Kong Baptist University, Hong Kong, China; ^2^International Medical Service Center, The First Affiliated Hospital of Shantou University Medical College, Shantou, China; ^3^School of Pharmacy, Chengdu University of Traditional Chinese Medicine, Chengdu, China

**Keywords:** Cathepsin K, osteoclast, bone resorption, osteoporosis, osteoarthritis, Cathepsin K inhibitor, cardiovascular diseases, lung fibrosis

## Abstract

Cathepsin K (CatK) is one of the most potent proteases in lysosomal cysteine proteases family, of which main function is to mediate bone resorption. Currently, CatK is among the most attractive targets for anti-osteoporosis drug development. Although many pharmaceutical companies are working on the development of selective inhibitors for CatK, there is no FDA approved drug till now. Odanacatib (ODN) developed by Merck & Co. is the only CatK inhibitor candidate which demonstrated high therapeutic efficacy in patients with postmenopausal osteoporosis in Phase III clinical trials. Unfortunately, the development of ODN was finally terminated due to the cardio-cerebrovascular adverse effects. Therefore, it arouses concerns on the undesirable CatK inhibition in non-bone sites. It is known that CatK has far-reaching actions throughout various organs besides bone. Many studies have also demonstrated the involvement of CatK in various diseases beyond the musculoskeletal system. This review not only summarized the functional roles of CatK in bone and beyond bone, but also discussed the potential relevance of the CatK action beyond bone to the adverse effects of inhibiting CatK in non-bone sites.

## Introduction

Lysosomal cysteine proteases, firstly discovered in the 20s of the twentieth century, are generally called cathepsins nowadays. In human, there are 11 members of cathepsins (cathepsin B, C, F, H, K, L, O, S, V, W, and Z), which are distinguished by their structures, catalytic mechanisms, and which proteins they cleave (Turk et al., 2001). This big family shares a common papain-like structural fold and a conserved active site Cys-Asn-His triad of residues (Vasiljeva et al., 2007). Among them, Cathepsin K (CatK) is predominantly secreted by activated osteoclasts to degrade collagen and other matrix proteins during bone resorption (Costa et al., 2011). Human CatK is a 329-amino-acid long protein consisting of an N-terminal 15-amino-acid long signal sequence, a 99-amino-acid long propeptide and a 215-amino-acid long catalytic unit (Bromme and Okamoto, 1995). From the structural aspect, CatK has the typical three-dimensional structure of a CatL like peptidase. The active site of CatK is a V-shaped cleft located at the top of the molecule and contains the catalytic diad cysteine–histidine. It can accept a Pro residue in the P2 position, which is correlated to the high content of Pro and hydroxyproline residues in collagen (McGrath et al., 1997; Novinec and Lenarcic, 2013). Since suppression of CatK activity could prevent bone resorption without perturbing bone formation (Bromme and Lecaille, 2009; Costa et al., 2011; Garber, 2016), it has become an attractive target for anti-resorptive drug development.

Many pharmaceutical companies are working on the development of selective inhibitors for CatK, most efforts focused on the cysteine thiol moiety of CatK with reactive electrophile “warheads” to reversibly inhibit or irreversibly inactivate its proteolytic activity (Bromme and Lecaille, 2009; Wijkmans and Gossen, 2011; Lu et al., 2018). Nevertheless, there is no FDA approved drug till now. Balicatib (AAE581) developed by Novartis terminated in its Phase II trial due to cutaneous lesions such as pruritus, skin rashes and rare morphea-like skin changes (Peroni et al., 2008; Runger et al., 2012). ONO-5334, a novel synthetic inhibitor of CatK, developed by Ono Pharmaceutical Co has passed its phase I and II clinical trials (Tanaka et al., 2017). But this project was recently terminated due to some market reasons. Odanacatib developed by Merck & Co. is the only CatK inhibitor candidate which demonstrated high therapeutic efficacy in patients with postmenopausal osteoporosis in Phase III clinical trials (McClung et al., 2019). Unfortunately, due to the undesirable adverse effects in non-bone tissues, i.e., higher incidence of cardio-cerebrovascular events vs. placebo group, the development of ODN was finally stopped. CatK was previously believed to be mainly secreted by osteoclasts and active at the bone resorption surfaces (Drake et al., 1996; Costa et al., 2011). However, it becomes clear at this point that the activity of CatK has far-reaching effects throughout various organs beyond bone. Therefore, it is essential to ascertain the role of CatK in bone and non-bone sites as well as in cell types besides osteoclasts. Here, we reviewed the functional role of CatK in bone and beyond bone. We also discussed the potential relevance of the CatK action beyond bone to the adverse effects of inhibiting CatK in non-bone sites.

## Role of CatK in Bone

### CatK and Bone Cells

The abundant expression of CatK instead of the other cathepsins was previously identified in osteoclasts (Drake et al., 1996), the unique bone cell type responsible for absorbing bone matrix in skeletal modeling and remodeling. It has been known that CatK expression is regulated by the receptor activator of nuclear factor κB ligand (RANKL)-RANK signaling (Troen, 2006), the critical signaling pathway of osteoclastogenesis. The activation of RANKL-RANK signaling pathway in osteoclast precursors stimulates the pro-osteoclastogenic transcriptional factor NFATc1 (nuclear factor of activated T cells) to initiate the transcription of CatK (Balkan et al., 2009). Many other factors, such as tumor necrosis factor-α (TNF-α), interleukins, vitamin D, and parathyroid hormone, could also stimulate CatK expression in osteoclasts (Troen, 2006). The CatK secretion could be regulated by the interaction between the E3 ubiquitin ligase Cbl and Phosphatidylinositol-3 Kinase (PI3K) in osteoclasts (Yu et al., 2019). In the process of bone resorption, CatK are secreted from mature osteoclast into the “sealing zone,” a dynamic actin-rich cell–matrix adhesion structure that defines the resorption area of bone (Takito et al., 2018), wherein the acidified milieu could dissolve the mineralized component of bone for exposing its organic matrix, which were subsequently degraded by CatK and other proteases. It is known that CatK could efficiently degrade type I collagen (Garnero et al., 1998; Kafienah et al., 1998), which constitutes approximately 90% of bone organic matrix, and extracellular matrix glycoprotein osteonectin (Bossard et al., 1996), which is among the remaining 10% constituent of bone organic matrix and is critical in supporting bone remodeling and maintaining bone mass (Delany et al., 2000). Impressively, CatK is the only osteoclast-secreted protease that is able to cleave both the triple helix and telopeptides of type I collagen fibers (Bromme and Okamoto, 1995; Garnero et al., 1998). CatK could also degrade type II collagen, the predominant matrix protein in cartilage (Kafienah et al., 1998). In addition, it was previously shown that CatK could cleave and activate the matrix-metalloproteinase-9 (MMP-9) (Christensen and Shastri, 2015), another protease necessary for bone matrix degradation. Importantly, the CatK deficient mice developed osteopetrosis while the CatK deficient osteoclasts were defective in resorbing demineralized bone (Gowen et al., 1999). Collectively, it could be concluded that CatK is indispensable for osteoclast-mediated bone resorption.

Although a previous immunoelectron microscopic study on mouse femur showed the negligible CatK expression in both osteoblasts and osteocytes (Yamaza et al., 1998), the other two important bone cell types, it is now known that CatK could be expressed and secreted by these two types of bone cells to exert its function of degrading bone matrix. The expression and secretion of CatK was found in human trabecular bone-derived osteoblasts from patients with fracture of femoral neck, which was thought to probably contribute to the maintenance of bone organic matrix and recycling of improperly processed type I collagen (Mandelin et al., 2006). On the other hand, it was recently found that osteocytes could also express and secrete CatK, which was required for the lactation-induced peri-lacunar resorption to guarantee the adequate amounts of calcium in milk for the skeletal development in offspring (Lotinun et al., 2019). In addition, it was demonstrated that mechanical loading could stimulate osteoblasts and osteocytes to express CatK whereby it could modulate modeling-based cortical bone formation by degrading periostin (Bonnet et al., 2017), the matricellular protein secreted by these two bone cell types essential for periosteal bone formation in response to mechanical loading (Bonnet et al., 2009). Intriguingly, a recent study has identified a population of periosteal stem cell (PSC) with CatK expression that could contribute to mediate intramembranous bone formation (Debnath et al., 2018). These CatK-expressing PSCs also possessed the capacity to differentiate into chondrocytes to mediate endochondral bone formation in fracture healing. However, the functional role of CatK in PSCs remains to be answered in future study.

### CatK and Skeletal Diseases

The CatK function has been linked with skeletal homeostasis ever since the discovery of the mutations in CatK gene that are responsible for pycnodysostosis (Gelb et al., 1996), a rare autosomal recessive disorder characterized by osteopetrosis, bone fragility, and decreased bone turnover. *In vitro* studies revealed that the Pycnodysostosis-related mutant CatK proteins were incapable of degrading type I collagen (Hou et al., 1999). Consistently, the urine levels of cross- linked N- and C-telopeptides of type I collagen (NTX and CTX), two bone resorption markers reflecting the degradation of type I collagen, were both decreased in the pycnodysostosis (Pycno) patients (Nishi et al., 1999), which suggests the low bone resorption activity in pycnodysostosis. Interestingly, the serum levels of Tartrate-resistent acid phosphatas (TRAP) levels, an osteoclast marker, and type I collagen carboxy-terminal propeptide (PICP) and osteocalcin, two bone formation markers reflecting bone martix synthesis, were all normal in Pycno patients (Nishi et al., 1999), suggesting that the generation of osteoclast and bone formation capacity were not affected by the lack of CatK activity in pycnodysostosis. In fact, all the CatK mutations identified in Pycno patients were loss-of-function mutations that appeared to eliminate its enzymatic activity (Hou et al., 1999; Schilling et al., 2007; Li et al., 2009; Bertola et al., 2010; Khan et al., 2010). The bone phenotype of pycnodysostosis was later reproduced in the CatK gene knockout mice, which developed mild osteopetrosis with increased trabecular and cortical bone mass due to impaired osteoclastic bone resorption (Saftig et al., 1998). In contrast, overexpression of CatK in mice resulted in the accelerated bone turnover (Kiviranta et al., 2001). Consistently, it was found that the serum CatK levels were significantly elevated in women with postmenopausal osteoporosis, while they were reduced after the patients were treated with bisphosphonates, the established anti-resorptive agents (Meier et al., 2006). Thus, all these preliminary findings lent support to the idea that targeting CatK could be a promising anti-resorptive strategy for osteoporosis. Thus far, a series of CatK inhibitors have been designed against the human CatK and developed, most of which have proved efficacious in suppressing osteoclastic bone resorption and preventing bone loss in the osteoporotic rodents or non-human primates in preclinical study. Moreover, several inhibitors have been evaluated in clinical trials to treat osteoporosis, which showed excellent efficacy on reducing bone resorption and improving trabecular bone mineral density (BMD) in osteoporotic patients. Unfortunately, despite the beneficial effect on bone, all these CatK inhibitors have currently been discontinued due to various adverse effects beyond bone in clinical trials (Drake et al., 2017).

It is noteworthy that a high bone-formation rate was also observed in the CatK-deficient (CatK^–^/^–^) mice (Li et al., 2006), while the bone formation remains normal in patients with pycnodysostosis (Nishi et al., 1999; Chavassieux et al., 2008). Impressively, unlike the other classes of anti-resorptive agents (Baron et al., 2011), CatK inhibitors generally do not perturb bone formation (Bromme et al., 2016; Drake et al., 2017). This unexpected phenomenon suggests the uncoupling of bone resorption and bone formation under the deficiency of CatK function. The available evidence from preclinical studies have demonstrated that either CatK deficiency or inhibition did not affect the osteoclastogenesis *in vitro* (Leung et al., 2011), whereas they could maintain or led to the increased osteoclast formation in multiple preclinical animal models *in vivo* (Pennypacker et al., 2009; Duong, 2012). Accordingly, these findings indicate that CatK inhibition, contrary to other anti-resorptive strategies, does not perturb osteoclast formation and survival that are required for the osteoblastic bone formation response during remodeling (Martin and Sims, 2005). From the molecular aspect, a study by Lotinun et al. (2013) found that the CatK-deficient osteoclasts would secrete more sphingosine-1-phosphate (S1P) to enhance osteoblastic bone formation. Moreover, it was recently shown by Xie et al. (2014) that inhibition of CatK could result in the increased number of preosteoclasts, which would facilitate the secretion of platelet-derived growth factor-BB (PDGF-BB) from preosteoclasts to induce the formation of CD31^hi^Emcn^hi^ vessel subtype (highly expressing CD31 and endomucin) for stimulating bone formation.

Arthritis is a collection of joint disorders affecting the articular cartilage, bone and periarticular tissues such as synovium due to the aberrant mechanical stimulation and/or inflammation. Osteoarthritis (OA) and rheumatoid arthritis (RA) are the most common forms of arthritis, of which the primary pathological feature is the progressive cartilage matrix degradation (Pap and Korb-Pap, 2015). CatK has been implicated in cartilage matrix degradation and OA, since type II collagen, the predominant matrix protein of cartilage, are the substrates of CatK (Kafienah et al., 1998; Mort et al., 2016), while chondroitin sulfates, the glycosaminoglycans (GAGs) abundant in cartilage matrix, could specifically increase the stability and collagenolytic activity of CatK (Li et al., 2000). A recent study further demonstrated that the excess mechanical stress loading could stimulate the CatK expression in human chondrocytes (Suzuki et al., 2020). Moreover, it was shown that the CatK expression in OA cartilage increased in relation to the severity of OA (Konttinen et al., 2002). In human normal cartilage, the CatK expression was only detected in chondocytes at the deep zone of cartilage near subchondral bone, while it was detected in chondrocytes at all layers especially the superficial layer with fissures at cartilage surface in human OA cartilage as well as in human OA synovial tissues (Konttinen et al., 2002; Kozawa et al., 2016). In accordance with these findings, the collagenase-generated cleavage neoepitopes of type II collagen were found to be abundant and extended at all layers of human OA cartilage but only limited in the pericellular area of chondrocyte in human normal cartilage (Dejica et al., 2012). More importantly, the CatK-deficient mice revealed the milder cartilage degradation after anterior cruciate ligament transection (ACLT) when compared with wildtype controls (Hayami et al., 2012), whereas the CatK transgenic mice spontaneously developed synovitis and cartilage degradation (Morko et al., 2005). Taken together, these findings suggest that the increased expression and collagenase activity of CatK could contribute to the cartilage matrix degradation in OA progression. Consequently, CatK is currently among the promising therapeutic target candidates for the development of disease-modifying osteoarthritic drugs. It is encouraging to learn that CatK inhibition could exert obvious chondroprotective effect in preclinical OA models (Connor et al., 2009; McDougall et al., 2010; Hayami et al., 2012; Lindstrom et al., 2018). Furthermore, a novel selective CatK inhibitor MIV-711 by Medivir was recently reflected to reduce bone remodeling and cartilage volume loss but have no impact on pain in OA patients in a Phase-IIa trial (Conaghan et al., 2019). Nevertheless, the structural benefits by CatK inhibition in OA should be further evaluated and confirmed in longer and larger trials.

Apart from OA, CatK was also involved in RA progression. The high CatK expression was detected in the synovial tissues, particularly in synovial fibroblast, from RA patients (Hummel et al., 1998), as well as in osteoclasts, articular cartilage and synovial tissue in arthritic joints of cynomolgus monkey with collagen-induced arthritis (CIA) (Tanaka et al., 2016), which implies the contribution of CatK to articular bone destruction in the affected joints in RA Further, it was shown that the serum CatK levels were increased in patients with active longstanding RA, which were significantly correlated with the severity of joint destruction (Skoumal et al., 2005). By genetic approach, it was found that CatK deficiency largely prevented the cartilage erosion and bone destruction and reduced the joint inflammation in mice with TNF-α-mediated arthritis (Hao et al., 2015). By pharmacological approach, several preclinical studies concordantly showed that inhibition of CatK could suppress the cartilage degradation as well as the systemic and local bone loss to prevent joint destruction in preclinical RA models (Asagiri et al., 2008; Svelander et al., 2009; Yamashita et al., 2018; Yamada et al., 2019). Interestingly, the efficacy of CatK inhibition on joint inflammation are contradictory among these studies. Those studies on CIA rodent models all revealed improvement in joint inflammation with CatK inhibition, whereas the study on CIA cynomolgus monkeys showed no impact on joint inflammation with CatK inhibition. This discrepancy maybe attributed to the different susceptibility and immune response to heterogenous collagen between rodents and non-human primates. Collectively, a detrimental role of CatK in RA progression could be concluded, which also suggests that targeting CatK could be a promising therapeutic strategy for RA.

Briefly, the roles of CatK in skeletal diseases are summarized in Table 1. Given the fundamental role in mediating bone and cartilage matrix degradation as well as the unique action to bone formation response after inhibition, CatK has become one of the most attractive therapeutic targets in bone in the past two decades. The fact is that most of the CatK inhibitors have failed in clinical trials due to adverse effects beyond bone (Bromme and Lecaille, 2009; Drake et al., 2017). Still, lessons learned from these failures not only extend our knowledge about the underlying biology of CatK and clinical efficacy of CatK inhibitors, but also prompt us to reconsider the CatK action in non-bone sites, which would be valuable for the design and development of novel CatK inhibition strategies.

**TABLE 1:** Role of CatK in bone.

**Tissues**	**Diseases**	**Functions**	**References**
Bone	Pycnodysostosis	Pycnodysostosis-related mutant CatK proteins were incapable of degrading type I collagen. All the CatK mutations identified in Pycno patients were loss-of-function mutations that appeared to eliminate its enzymatic activity.	Gelb et al., 1996; Hou et al., 1999; Nishi et al., 1999
	Postmenopausal osteoporosis	The serum CatK levels were significantly elevated in women with postmenopausal osteoporosis, while they were reduced after the patients were treated with bisphosphonates, the established anti-resorptive agents.	Meier et al., 2006
	OA	The increased expression and collagenase activity of CatK could contribute to the cartilage matrix degradation in OA progression. CatK is currently among the promising therapeutic target candidates for the development of disease-modifying osteoarthritic drugs.	Konttinen et al., 2002; Dejica et al., 2012; Kozawa et al., 2016
	RA	The serum CatK levels were increased in patients with active longstanding RA, which were significantly correlated with the severity of joint destruction. CatK deficiency largely prevented the cartilage erosion and bone destruction and reduced the joint inflammation in mice with TNF-α-mediated arthritis. Inhibition of CatK could suppress the cartilage degradation as well as the systemic and local bone loss to prevent joint destruction in preclinical RA models.	Skoumal et al., 2005; Asagiri et al., 2008; Svelander et al., 2009; Hao et al., 2015; Yamashita et al., 2018; Yamada et al., 2019

## Role of CatK Beyond Bone

### Central Nervous System (CNS)

CatK has been found to be unevenly and widely distributed in adult rat and human brain especially in multiple neurons as well as in glial cells (Bernstein et al., 2007). Moreover, a previous study by Brix’s group revealed a wide range of both molecular and cellular changes in the CNS after ablation of the CatK gene in mice (Dauth et al., 2011). It is noteworthy that an imbalance of the proteolytic network of cysteine cathepsins was found in the brains of CatK-deficient mice. Particularly, when compared with the wildtype mice, the amounts of CatB and CatL proteins in the cortex were decreased while their enzyme activities were unaltered in CatK-deficient mice. Meanwhile, the amounts of CatB and CatL proteins were increased in the striatum/mesencephalon and hippocampus in CatK-deficient mice, respectively, while their enzyme activities decreased together with the increased protein amounts of cystatin C, the endogenous cysteine protease inhibitor, in the same brain regions. These interesting findings indicate that CatK deficiency could trigger the compensative expression of CatB and CatL in brain, while the upregulated cystatin C may serve as a self-feedback mechanism in response to such compensation. Importantly, the dysregulation of CatB and CatL in cerebral cortex has been implicated with dramatic consequences in CNS, including neurodegeneration and brain atrophy (Felbor et al., 2002; Houseweart et al., 2003). Moreover, CatK-deficient mice exhibited reduced anxiety levels and both short- and long-term learning and memory deficits as compared to the wildtype mice (Dauth et al., 2011). Although two later studies by the same group have excluded the possible influence of CatK’s function in thyroid and astrocytes on the brain phenotype of CatK-deficient mice (Dauth et al., 2012, 2019), the underlying molecular mechanism responsible for the CNS dysfunction in CatK-deficient mice remains elusive. Cumulatively, it indicates that the existence of CatK should be extremely vital for the development and metabolism of CNS.

The involvement of CatK has been found in various CNS diseases, including stroke, cerebral aneurysm (CA), chronic subdural hematoma (CSDH), and schizophrenia. Zhao et al. (2019) have reported a protective role of CatK in acute ischemic stroke (AIS). The recombinant tissue plasminogen activator (rtPA) is the only approved drug for the standard reperfusion treatment for AIS but could aggravate blood-brain barrier BBB disruption and increase the risk of life-threatening hemorrhagic transformation (HT) (Wardlaw et al., 2013; Khurana et al., 2017; Pena et al., 2017). The study by Zhao et al. (2019) found that CatK-deficient mice had the higher risks of HT, cerebral oedema, blood-brain barrier (BBB) disruption, neurological deficits, and infarct volume after rtPA-treated cerebral ischemia comparing with the wild types. At the molecular levels, they observed the upregulation of vascular endothelial growth factor (VEGF) and downregulation of Akt/mTOR pathway in the ischemic brain of CatK-deficient mice, which were thought to contribute to the BBB leakage in the acute ischemic brain (Zhang et al., 2000, 2017; Greenberg and Jin, 2013). Although a protective role of CatK on BBB integrity has been proposed in this study, the underlying mechanism is largely unknown and requires further study. Nevertheless, it hints that the inhibition of CatK could pose higher risk of rtPA-induced HT in patients with AIS.

The extensive breakdown of extracellular matrix (ECM) in arterial wall is a prominent pathological feature of cerebral aneurysm (CA) (Penn et al., 2011). The study by Aoki et al. (2008) attributed this aberrant change to the imbalance between cysteine cathepsins, including CatK, CatB, and CatS, and their endogenous inhibitor Cystatin C in CA development. It was found that the expression of CatK and the other two cysteine cathepsins were upregulated mainly in the late stage of CA, whereas the Cystatin C expression was downregulated with the progression of experimental CA in rats. Impressively, administration of NC-2300, an inhibitor of most of cysteine proteases, could prevent CA progression in rats by inhibiting ECM degradation in aneurysmal walls caused by cysteine cathepsins. These results suggest that inhibition of CatK might be beneficial for preventing CA progression. Intriguingly, the unusual imbalance between CatK and Cystatin C was also found to be associated with the process of chronic subdural hematoma (CSDH), another pathological conditions of CNS after head injury, according to the research by Tsutsumi et al. (2017) on a group of 42 patients with symptomatic CSDH. The results showed that both the CatK and Cystatin C levels were concentrated in CSDH and intracranial cerebrospinal fluid (CSF). However, in peripheral venous blood of CSDH patients, the CatK levels were increased while the Cystatin C levels were decreased when compared with normal controls. The immunohistochemistry staining further revealed the diffuse CatK expression in the outer membrane of CSDH (Tsutsumi et al., 2017). This study suggests that CatK is highly relevant with the development of CSDH.

It was previously found that the CatK gene were among the very few genes that were linked to schizophrenia with various antipsychotic treatment (Ko et al., 2006), wherein its expression in rodent brain striatum was downregulated by the psychotropic substance amphetamine but was upregulated by typical and atypical neuroleptic. Consistently, Lendeckel et al. reported in a later study showing that the CatK expression was markedly upregulated in postmortem brains of patients suffering from schizophrenia who received long-term treatment with neuroleptics (Bernstein et al., 2007). By immunohistochemistry, they further identified that CatK was largely colocalized with enkephalinergic and endorphinergic innervated structures in hypothalamic nuclei (Lendeckel et al., 2009). Interestingly, they also observed a dose-dependent increase of CatK mRNA expression in dopamine receptor-expressing SH-SY5Y cells in response to the treatment of the neuroleptic agent haloperidol (Lendeckel et al., 2009), which reinforced the previous findings by Ko et al. (2006). They further demonstrated that the upregulated cerebral CatK expression by neuroleptic treatment might contribute to the altered opioid levels in brains of schizophrenics probably through processing β-endorphin to release met-enkephalin (Lendeckel et al., 2009).

### Cardiovascular System

The endocardial CatK expression is regulated by RANKL (receptor activator of NFκB ligand)/RANK-NFATc1 signaling pathway (Lange and Yutzey, 2006). Although only a small amount of CatK was detected in normal heart and artery tissues (Inaoka et al., 1995; Sukhova et al., 1998), its expression was significantly upregulated in failing heart, abdominal aortic aneurysms (AAA) lesion and atherosclerotic lesions (Sukhova et al., 1998; Shi et al., 1999; Lutgens et al., 2006). Consistently, the existing evidence from a series of studies on the genetic CatK knockout mouse reveal that CatK is not essentially required for the organogenesis and postnatal development of cardiovascular system (Lange and Yutzey, 2006), but it is actively involved in the progression of various cardiovascular diseases (Lutgens et al., 2007).

The link between CatK and cardiac dysfunction has been extensively studied. Zhao and colleagues previously found that the elevated serum CatK levels were closely associated with the presence of chronic heart failure (CHF) (Zhao et al., 2015). They showed that patients with CHF who showed a low left ventricular ejection fraction (LVEF) had significantly higher serum CatK levels than those who showed a high LVEF. Consistently, in another study by Hua et al. (2013a) they detected the high CatK expression in human hearts of end-stage dilated cardiomyopathy. On the other hand, the Nair’s group has reported serial studies on CatK knockout mice to authenticate the essential contribution of CatK to cardiac dysfunction, including the obesity-associated cardiac hypertrophy (Hua et al., 2013b), the pressure overload–induced cardiac hypertrophy (Hua et al., 2013a), the diabetes-induced cardiac anomalies (Guo et al., 2017a), and the aging-induced cardiac dysfunction (Hua et al., 2015). They reported several benefits of CatK deficiency to the heart including : (1) inhibiting the expression of cardiac hypertrophic proteins and apoptotic markers and partly reversing the impaired cardiomyocyte contractility associated with high-fat diet (Hua et al., 2013b), (2) attenuating the pressure overload-induced upregulation of mammalian target of rapamycin and extracellular signal-regulated kinases (ERK) signaling cascades and partly resolving the pressure overload-induced cardiac hypertrophy (Hua et al., 2013a), (3) reducing the cardiac oxidative stress and calcineurin/NFAT signaling and mitigating the cardiac dysfunction in streptozotocin-induced diabetes (Guo et al., 2017a), and (4) ameliorating the aging-related decline in cardiac function via suppressing caspase-dependent and caspase-independent cardiomyocyte apoptosis (Hua et al., 2015). Moreover, in another study on the cardiomyocyte-specific CatK-deficient mouse model by the same group, they showed that the doxorubicin-induced cardiotoxicity could be ameliorated by cardiomyocyte-specific ablation of CatK (Guo et al., 2018). Collectively, these serial studies hint the crucial role of CatK in cardiac dysfunction. Intriguingly, a recent study by Fang et al. (2019) showed that the plasma CatK levels were significantly increased in patients with coronary heart diseases (CHD) particularly in those with acute myocardial infarction (MI) when compared to non-CHD controls. They further detected the elevated CatK expression in heart from the post-MI mouse, which mainly localized to cardiomyocytes, ECs, fibroblasts, macrophages and CD4^+++^ T cells as well. Surprisingly, contrary to the aforementioned protective role of CatK deficiency on cardiac function (Hua et al., 2013a, b, 2015; Guo et al., 2017a, 2018), they found that the CatK-deficient mice exhibited worsen post-MI cardiac function along with increased collagen deposition and fibrosis, enhanced cardiac cell death, and reduced cardiac cell proliferation than the wildtype controls. Moreover, they showed that CatK deficiency or inhibition increased cardiomyocyte death, whereas CatK inhibition suppressed CD4+ T-cell and macrophage death *in vitro*. These findings in turn hint a protective role of CatK on the cardiac function after MI. In addition, a research group from Japan studied the link between CatK and atrial fibrillation (AF) (Fujita et al., 2013). They found that the plasma CatK levels were higher in patients with persistent AF than those with paroxysmal AF. Moreover, the atrial CatK expression and activity were found to be also increased in the rabbits with tachypacing-induced AF, which could be attributed to the activation of Ang II (angiotensin II)/AT1R (angiotensin type 1 receptor) signaling pathway in the atrium with AF. Although the atrial CatK expression seems to be closely associated with the presence of AF, it requires further mechanistic studies to determine the effect of CatK deficiency or inhibition on AF.

Apart from cardiac dysfunction, CatK has been implicated in the pathogenesis of other cardiovascular disorders including AAA, coronary artery diseases and atherosclerosis. The concurrent overexpression of CatK and CatS was previously identified in the human AAA lesions (Shi et al., 1999). A later study by Sun and colleagues further confirmed that CatK could play an essential role in AAA formation (Sun et al., 2012). They found that CatK knockout mice were protected from elastase perfusion-induced experimental AAA. Mechanistically, it was shown that CatK could promote T-cell proliferation, vascular SMC apoptosis, and elastin degradation to contribute to AAA formation. Cheng et al. (2013) studied the relationship between plasma circulating CatK and the prevalence of coronary artery disease (CAD), by which they showed that CatK level was an independent predictor of CAD. In this study, they found that patients with CAD had higher circulating CatK levels than the non-CAD controls. The CatK levels were positively correlated with the ratios of ICTP (cross linked carboxy-terminal telopeptide of collagen type I) /I-PINP (intact procollagen type I N-terminal propeptide), the collagen turnover-related index, indicating that the elevated circulating CatK levels were closely associated with the increased collagen turnover in CAD patients. Interestingly, among the CAD patients, the individuals with acute coronary syndrome had higher CatK levels than those with stable angina pectoris. More importantly, the CatK levels were found to be correlated positively with the percent plaque volumes and inversely with the percent fibrous volumes of coronary artery by intravascular ultrasound. These clinical findings were in line with the previous histopathological findings in human atheroma specimens showing that CatK expression was remarkably upregulated in the atherosclerotic lesions of human aorta (Sukhova et al., 1998; Lutgens et al., 2006). Specifically, the CatK expression was detected in the intima and medial SMCs of early human atherosclerotic lesions and in macrophages and SMCs of the fibrous cap in advanced atherosclerotic plaques (Sukhova et al., 1998). All these findings indicate the possible involvement of CatK in atherosclerosis. The role of CatK on the progression of atherosclerotic plaque was further investigated in the study by Lutgens and colleagues using the atherosclerosis-prone mouse model with CatK deficiency (CatK^–^/^–^apoE^–^/^–^ mice) (Lutgens et al., 2006). They found that the deficiency of CatK resulted in the reduced number of advanced lesions as well as decreased individual advanced plaque area but increased number of initial plaques in the CatK-deficient apoE^–^/^–^ mice as compared with the CatK-intact apoE^–^/^–^. Moreover, the atherosclerotic plaques were highly fibrotic in CatK-deficient apoE^–^/^–^ mice, which characterizing a stable plaque phenotype. On the other hand, Platt et al. observed the increased expression and activity of CatK in mouse aortic endothelial cells (MAEC) exposed to proatherogenic oscillatory shear (OS) than the MAECs exposed to atheroprotective, unidirectional laminar shear stress (LS) (Platt et al., 2007). They further showed that knocking down CatK with small-interfering RNA (siRNA) could decreased OS-dependent elastase and gelatinase activities in MAECs. Similarly, another recent study reported that genetic knockout of CatK could mitigate the calcification, migration and proliferation of mouse aortic vascular SMCs (Guo et al., 2017b), which may contribute to the vascular calcification and intimal thickening in atherosclerosis. Collectively, these findings suggest that CatK deficiency may have a protective role in atherosclerosis by increasing plaque fibrosis and decreasing the aberrant arterial wall remodeling. Interestingly, in the aforementioned study by Lutgens et al. (2006) they also showed that CatK deficiency in apoE^–^/^–^ mice simultaneously accelerated the formation of macrophage foam cells, the featured cell type occupying much of the lesion volume in early to intermediate atherosclerotic lesion that could lead to the progression of atherosclerosis (Rader and Pure, 2005). Both the scavenger receptor (SR)-mediated uptake of oxidized low-density lipoprotein (OX-LDL) and the storage of cholesterol esters in macrophages, as well as the lysosomal size of macrophage foam cells, were found to be increased in the absence of CatK (Lutgens et al., 2006). In addition, the CatK-deficient apoE^–^/^–^ mice in this study showed a trend toward increased serum cholesterol, LDL cholesterol, and triglyceride levels and decreased HDL cholesterol levels (Lutgens et al., 2006), while these changes in serum lipid profile are generally considered as the clinical pro-atherogenic factor (Linton et al., 2000). Therefore, these findings not only hint a potential role of CatK in lipid metabolism, but also indicate that CatK deficiency could aggravate the formation of macrophage foam cells, which may affect the plaque stability.

### Respiratory System

It is known that the lysosomal cysteine proteases play a crucial role in ECM remodeling, immunoregulation and surfactant protein processing in the lung (Buhling et al., 2004). As one of the most potent cysteine proteases, CatK has been linked with various diseases of the respiratory system. The aberrant CatK activity is closely associated with the inadequate collagen turnover in the lung.

The expression of CatK was detected in the bronchial and alveolar epithelial cells as well as alveolar macrophages in the normal lung (Buhling et al., 1999; Haeckel et al., 1999; Buhling et al., 2001). In fact, CatK plays a pivotal role in pulmonary homeostasis through collagen cleavage, which is required for the airway structural integrity (Bühling et al., 2004; Zhang et al., 2011). A previous study on CatK-deficient mice demonstrated that CatK could partly regulate mouse airway development (Zhang et al., 2011). Meanwhile, TGF-β1 was proven as an efficient substrate of CatK. This finding echoed the previous finding on the inverse correlation between the expression of CatK and TGF-β1 in lung tissues of mice with silica-induced lung fibrosis (van den Brule et al., 2005). Collectively, it indicates that the interaction between CatK and TGF-β1 might be necessary for preventing the excessive TGF-β1-driven airway remodeling, which is important for the airway development and lung homeostasis. Bühling et al. (2004) reported a protective role of CatK in lung fibrosis. They showed that CatK-deficient mice developed more pulmonary fibrosis than wildtype controls after bleomycin induction. The pulmonary fibroblast from CatK-deficient mice showed decreased cathepsin-mediated collagenolytic activity as compared with those from wildtype mice, whereas pulmonary fibroblast from patients with lung fibrosis exhibited enhanced CatK activity. Interestingly, they further showed that, comparing with nonfibrotic lung tissues, CatK expression was significantly upregulated in the fibrotic lung tissues from mice and patients with pulmonary fibrosis, which could be dominantly contributed by the excessive CatK expression in pulmonary fibroblasts but not in bronchial epithelial cells and alveolar macrophages that were previously considered as the major cell type with CatK expression in lung (Buhling et al., 1999, 2001; Haeckel et al., 1999). The upregulated CatK expression in the fibrotic lung may reflect the self-defense mechanism in response to lung fibrosis.

Despite the protective role of CatK on lung fibrosis is univocal, the over-expression of CatK could be harmful in some other lung diseases. For example, lung emphysema, of which cigarette smoking is the major cause, was closely associated with CatK over-expression in lung (Zheng et al., 2000; Golovatch et al., 2009). The activity of CatK instead of MMPs was found to be upregulated in the lung tissues of guinea pig with cigarette smoke-induced emphysema, which may contribute to the remodeling of the lung ECM with disease progression (Golovatch et al., 2009). Additionally, a recent study done by Kubler et al. (2016) reported the negative impact of CTSK over-expression in active pulmonary tuberculosis (TB). It was observed that CatK was most abundantly expressed in cavitary lesion, which was associated with the collagen turnover of cavitation in a rabbit model of TB. They also found the increased plasma CatK levels in patients with TB when compared with healthy controls, which, as they suggested, was a feature of active pulmonary tuberculosis.

CatK has also been connected with some of the lung tumors. It was found that CatK was diffusely and strongly expressed in pulmonary perivascular epithelioid cell tumor and it maybe a potential biomarker for identification of such kind of disease (Calio et al., 2018). In addition, the expression of CatK was restricted in the LAM cells in pulmonary lymphangioleiomyomatosis (LAM), a rare disease in which LAM cells and fibroblasts form lung nodules (Chilosi et al., 2009). Dongre et al. (2017) recently reported that LAM nodule-derived CatK activity, which was thought to contribute to cyst formation and tissue damage in lung (Chilosi et al., 2009), was largely dependent on the interactions between LAM cells and lung fibroblasts.

### Other Organs and Systems

CatK could play a unique role in autoimmune diseases. Researchers have previously showed that either genetic deficiency or pharmacological inhibition of CatK could suppress the autoimmune inflammation in animals with experimental autoimmune encephalomyelitis and adjuvant-induced arthritis and lupus, respectively (Asagiri et al., 2008; Zhou et al., 2017). In the study by Takayanagi’s group, they demonstrated that the lack of CatK activity could affect the innate immune response to pathogen DNA by compromising Toll-like receptors 9 (TLR9) signaling in dendritic cells (DCs), leading to the attenuated induction of T helper 17 cells without affecting the antigen-presenting ability of DCs (Asagiri et al., 2008). In another study by Shi’s group, they showed that CatK could contribute to autoimmune inflammation by regulating TLR7 expression, proteolytically processing TLR7, reducing the T regulatory cells (Tregs) numbers, and suppressing Treg immunosuppression activity against T effector cells (Zhou et al., 2017).

CatK is also involved in the physiological function of thyroid. It is expressed by the thyroid epithelial cells and secreted into the follicular lumen for mediating the extracellular proteolysis of the prohormone thyroglobulin (TG), which is the essential process for the thyroxine liberation (Tepel et al., 2000; Friedrichs et al., 2003; Jordans et al., 2009). Interestingly, it was found that serum CatK levels were increased by a suppressive L-thyroxine therapy but negatively correlated with aging in patients with differentiated thyroid cancer, although reasons account for this phenomenon remains unclear (Mikosch et al., 2008). On the other hand, the osteoclast-like multinucleated giant cells (MGCs) with CatK-expression were previously identified in the tumor mass of patients with anaplastic carcinoma of the thyroid gland (ACT), which were postulated to contribute to the invasive behavior of this tumor (Gaumann et al., 2001).

The CatK expression was also detected in white adipose tissue (Soukas et al., 2000; Chiellini et al., 2003), and it was markedly increased in the overweight/obese mice and peoples as compared to their controls with normal weight (Xiao et al., 2006; Yang et al., 2008). Previous studies have elegantly demonstrated that CatK could play a critical role in adipogenesis, since its expression was detected in pre-adipocytes and gradually upregulated during adipocyte differentiation (Chiellini et al., 2003), while the adipogenesis was enhanced by CatK overexpression but retarded after CatK deficiency (Funicello et al., 2007; Yang et al., 2008). In addition, body weight gained after high-fat diet was remarkably decreased in mice with either genetic knockout or pharmacological inhibition of CatK when compared with the controls (Yang et al., 2008). Moreover, the CatK-deficient mice also revealed a higher lipolytic rate in young age, an increased rate of free fatty acid oxidation after high-fat diet, and the altered serum cholesterol profiles upon the apolipoprotein E knockout, respectively, when compared with wildtype controls (Lutgens et al., 2006; Funicello et al., 2007). These findings implied the role of CatK in lipid homeostasis and metabolism.

On the other hand, the activities of CatK in skin areas is conditional. CatK gene was not detectable under normal conditions (Runger et al., 2007), but it was strongly expressed under certain circumstances, such as inflammation, scar formation and other cell-rich fibrosing processes (Quintanilla-Dieck et al., 2009). It was found that CatK expression was especially prominent in young scars and reduced with time (Quintanilla-Dieck et al., 2009). In addition to scar formation, a vital role of CatK was also suggested in primary melanomas and cutaneous melanoma metastases (Quintanilla-Dieck et al., 2008). Besides, the upregulation of CatK in the psoriatic lesions of patients with psoriasis was documented by Hirai et al. (2013). Coincidently, the upregulated CatK was found to be associated with oral squamous cell carcinoma (OSCC), in which CatK may be served as a predictive biomarker according to the study by Leusink et al. (2018).

Collectively, the roles of CatK beyond bone are summarized in Table 2. To sum up, based on the currently available evidence from CatK-deficient mouse model, it appears that the lack of CatK activity could bring benefits to the heart and vessels. However, results from the Phase III clinical trial of odanacatib have recorded a significantly higher incidence of stroke, more episodes of new or recurrent atrial fibrillation or flutter and more cases of cardiovascular death in the odanacatib group as compared with placebo group (McClung et al., 2019), which highlight the cardiovascular adverse effects associated with CatK inhibition. It remains to be answered whether these cardiovascular adverse effects are attributable to the potential off-target of odanacatib or the direct inhibition of CatK in cardio-cerebrovascular tissues. Nevertheless, the inconsistency of cardiovascular impact between CatK knockout and CatK inhibition remind us that inactivation of this protease may not necessarily mimic the consequences of gene deficiency. Since inhibition of CatK with odanacatib treatment could result in the elevated CatK expression and accumulation in osteoclasts *in vitro* (Leung et al., 2011), it is plausible that CatK inhibitor could also induce the upregulation of CatK in cardio-cerebrovascular cells, which is thought to be detrimental to heart and vessels, and thus, worth further investigation. On the other hand, another CatK inhibitor Balicatib were terminated in Phase II trials due to cutaneous lesions such as pruritus, skin rashes and rare morphea-like skin changes as reported (Peroni et al., 2008; Runger et al., 2012). These dermatologic adverse events were thought to be attributed to the lysosomotropism property of this basic compound, which potentially led to the off-target inhibition of cathepsins B, L, and S expressed in skin fibroblasts. This failure in turn alerts us the importance of designing the highly selective CatK inhibitor to avoid off-target side effects.

**TABLE 2:** Role of CatK beyond bone.

**Tissues**	**Disease**	**Functions**	**References**
Central nervous system	AIS	CatK is required for maintaining the BBB integrity while the absence of CatK activity might result in higher risk of rtPA-induced HT after cerebral ischemia.	Zhao et al., 2019
	CA	Inhibiting CatK could inhibit the excessive ECM degradation, and thus might be beneficial for preventing CA progression.	Aoki et al., 2008
	CSDH	The CatK levels were increased while the Cystatin C levels were decreased when compared with normal controls, suggesting that CatK is highly relevant with the development of CSDH.	Tsutsumi et al., 2017
	Schizophrenia	The CatK expression was markedly upregulated in postmortem brains of patients suffering from schizophrenia who received long-term treatment with neuroleptics. This upregulation of CatK might contribute to the altered opioid levels in brains of schizophrenics probably through processing β-endorphin to release met-enkephalin.	Ko et al., 2006; Lendeckel et al., 2009
Cardiovascular system	Cardiac dysfunction	CatK deficiency are beneficial to cardiac dysfunction, including the obesity-associated cardiac hypertrophy, the pressure overload–induced cardiac hypertrophy, the diabetes-induced cardiac anomalies and the aging-induced cardiac dysfunction.	Hua et al., 2013a, b; Hua et al., 2015; Guo et al., 2017a
	MI	The CatK-deficient mice exhibited worsen post-MI cardiac function along with increased collagen deposition and fibrosis, enhanced cardiac cell death, and reduced cardiac cell proliferation than the wildtype controls.	Fang et al., 2019
	Atherosclerosis	The deficiency of CatK resulted in the reduced number of advanced lesions as well as decreased individual advanced plaque area but increased number of initial plaques in the CatK-deficient apoE^–^/^–^ mice as compared with the CatK-intact apoE^–^/^–^.	Lutgens et al., 2006
Respiratory system	Lung fibrosis	The pulmonary fibroblast from CatK-deficient mice showed decreased cathepsin-mediated collagenolytic activity as compared with those from wildtype mice, whereas pulmonary fibroblast from patients with lung fibrosis exhibited enhanced CatK activity.	Bühling et al., 2004
Other organs and systems	Autoimmune diseases	The lack of CatK activity could affect the innate immune response to pathogen DNA by compromising TLR9 signaling in DCs, leading to the attenuated induction of T helper 17 cells without affecting the antigen-presenting ability of DCs.	Asagiri et al., 2008
	Physiological function of thyroid	CatK is expressed by the thyroid epithelial cells and secreted into the follicular lumen for mediating the extracellular proteolysis of the prohormone TG, which is the essential process for the thyroxine liberation.	Tepel et al., 2000; Friedrichs et al., 2003; Jordans et al., 2009
	Overweight/obese	The adipogenesis was enhanced by CatK overexpression but retarded after CatK deficiency. Body weight gained after high-fat diet was remarkably decreased in mice with either genetic knockout or pharmacological inhibition of CatK when compared with the controls.	Funicello et al., 2007; Yang et al., 2008

## Conclusion

Currently, CatK was among the most attractive targets for anti-osteoporosis drug development. This cysteine protease bears important functions in mediating bone resorption. However, the activity of CatK has far-reaching effects throughout various organs besides bone. The fact is that CatK not only demonstrates vastly distinct functional roles beyond bone, but also involves in various diseases beyond the musculoskeletal system. Nevertheless, the exact role of CatK and the underlying mechanisms in these diseases are not well-elaborated. Therefore, more in-depth mechanistic studies are urgently required to delineate the critical roles of CatK in these diseases. In another word, the sophisticated roles of CatK in various diseases beyond the musculoskeletal system would in turn pose the higher risk of adverse effects in non-bone sites after inhibiting CatK. Thus, researchers should be alerted to these risks when CatK inhibitors are developed as anti-osteoporosis drugs, while physicians should be more cautious when CatK inhibitors are prescribed for anti-osteoporosis treatment in patients with or with increased risk of developing those diseases. On the other hand, aptamer-drug conjugates (ApDC) have emerged over the past decades as a class of potential targeting agents to improve the efficacy of the traditional chemical compounds and overcome their potential side effect in off-target organs (Chen et al., 2017). Therefore, as an alternative strategy, it would be desirable to design and develop the novel “smart” CatK inhibitor chemically conjugated with bone-targeted aptamer (Zhang et al., 2012; Liu et al., 2015), by which it would facilitate the CatK inhibitor targeting bone to reduce its exposure in non-bone sites so as to prevent the potential adverse effects beyond bone.

## Author Contributions

RD and ZW did literature searching and prepared the manuscript. HC, JLu and AL helped in revising the manuscript. JLiu and GZ conceived the review topic and wrote most of the manuscript.

## Conflict of Interest

The authors declare that the research was conducted in the absence of any commercial or financial relationships that could be construed as a potential conflict of interest.
